# Supplementary material for: A scaling approach to estimate the age-dependent COVID-19 infection fatality ratio from incomplete data
Source: PLoS One. 2021 Feb 17;16(2):e0246831. doi: 10.1371/journal.pone.0246831 (PMC7888669; doi:10.1371/journal.pone.0246831)
Supplement: S1 Table — In the last column, we detail the Figures and Tables generated with these data. All these data are freely available for scientific use at the INED’s website: https:/dc-covid.site.ined.fr/fr/donnees/ and for free use at the datadista GitHub: https://github.com/datadista/datasets/tree/master/COVID%2019. (PDF) [file pone.0246831.s001.pdf]

Country	dates	Origin of the data (from INED)	Data details	Demographic sources	Data used for
Spain	21/05/2020	Ministerio de Sanidad, Consumo y Bienestar Social (MSCBS)	Cumulative deaths with confirmed COVID-19 infection occurred in hospitals or elsewhere	Instituto Nacional de Estadística (INE) 01/07/2019	Figs. 3,4 and 6B and Tables S2, 1, and 2.
	21/05/2020	MSCBS from datadista database	Confirmed cases, hospitalizations, deaths occurred in hospitals or elsewhere, and entries in ICU	”	Figs. 5A
	21/05/2020	Red Nacional de Vigilancia Epidemiológica (RENAVE)	Deaths occurred in hospitals or elsewhere	”	Figs. 5B
Portugal	19/05/2020	Serviço Nacional de Saúde, SNS-Direção Geral da Saúde, DGS	Cumulative deaths with confirmed COVID-19 infection	Eurostat, © European Union, 1995-2020	Figs. 3 and 4 and Tables S2, 1, and 2.
Norway	20/05/2020	Folkehelseinstituttet, "COVID-19 Dagsrapport"	Cumulative deaths in hospitals and in other health institutions (nursing homes, etc) and homes	Norway Statistics, Table 07459: "Population, by age and sex, 1986 - 2020"	Figs. 3 and 4 and Tables S2, 1, and 2.
Netherlands	21/05/2020	Rijksinstituut voor Volksgezondheid en Milieu	Cumulative deaths with confirmed COVID-19 infection in hospitals and elsewhere	CBS Statistics Netherlands 01/01/2019	Fig 3A.
South Korea	15/04/2020	Korea Centers for Disease Control & Prevention (KCDC)	Cumulative deaths with confirmed COVID-19 infection	KOSIS (Korean Statistical Information Service) statistical database 23/04/2020.	Figs. 3 and 4 and Tables S2, 1, and 2.
Italy	18/05/2020	Italian National Institute of Health (Istituto superiore di sanità - ISS); Daily Infographics,	Cumulative deaths	Italian National Institute of Statistics (Istat) 01/01/2019	Figs. 3 and 4 and Tables S2, 1, and 2.
Germany	21/05/2020	Robert Koch-Institut (RKI)	Cumulative deaths with confirmed COVID-19 infection	Eurostat, © European Union, 1995-2020, 10/04/2020.	Figs. 3 and 4 and Tables S2, 1, and 2.
France	21/05/2020	Data are communicated daily by Public Health France (SpF) to the French Institut for Demographic Studies (INED)	Cumulative deaths with confirmed COVID-19 infection occurred in hospitals	L'Institut national de la statistique et des études économiques (Insee)	Figs. 3 and 4 and Tables S2, 1, and 2.
	22/03 to 21/05/2020	”	”	”	Figs. 1˙
England and Wales	22/05/2020	Office for National Statistics (ONS). Deaths registered weekly in England and Wales.	Cumulative deaths (COVID-19 was mentioned on the death certificate) occurred in hospitals or elsewhere.	Office for National Statistics (ONS) 30/06/2018	Figs. 3 and 4 and Tables S2, 1, and 2.
England	22/05/2020	National Health Service (NHS)	Cumulative deaths with confirmed COVID-19 infection occurred in hospitals.	Office for National Statistics (ONS) 30/06/2018	Fig 2.
Denmark	20/05/2020	Statens Serum Institut (SSI)	Cumulative deaths with confirmed COVID-19 infection	Statistics Denmark 01/01/2020	Figs. 3 and 4 and Tables S2, 1, and 2.

S1 Table. Source and details of the age-distributed data (by country) used in the analysis. In the last column, we detail the Figures and Tables generated with these data. All these data are freely available for scientific use at the INED's website: `https:/dc-covid.site.ined.fr/fr/donnees/` and for free use at the datadista GitHub: `https://github.com/datadista/datasets/tree/master/COVID%2019`.
