# Supplementary material for: A scaling approach to estimate the age-dependent COVID-19 infection fatality ratio from incomplete data
Source: PLoS One. 2021 Feb 17;16(2):e0246831. doi: 10.1371/journal.pone.0246831 (PMC7888669; doi:10.1371/journal.pone.0246831)
Supplement: S2 Table — We give the values extracted from the collapse of Fig 3B: the increase of the mortality with age (proportional to the uniform fatality ratio f^α) and the number of infections in each country with respect to the number of infections in Spain IC/ISpain equal to the collapsing constant D(C). The relative scaling of the mortality above 70 years old is expected to be significantly underestimated. Errors are obtained using the boostrap method at 95% of confidence. The errors of D(C) are only the statistical errors extracted from the data collapse, they do not include the systematic error associated to the different policies of death counting the different countries which would be much larger, we try to give a better estimate below. (PDF) [file pone.0246831.s002.pdf]

Scaling relations			
age group	$\propto \hat{f}_\alpha$	Country	$\mathcal{D}(\mathcal{C}) = I_\mathcal{C}/I(\text{Spain})$
0-9	28(9)	Spain	1.0(0)
10-19	51(14)	Portugal	0.045(2)
20-29	21(4)×10	Norway	0.0076(4)
30-39	60(10)×10	Korea	0.014(2)
40-49	18(3)×10^2	Italy	1.1(2)
50-59	63(8)×10^2	Germany	0.27(2)
60-69	21(1.6)×10^3	France	0.96(8)
70-79	69(7)×10^3	England	1.48(2)
80-89	22(3)×10^4	Denmark	0.02(-)
90+	5.6(16)×10^5		

S2 Table. Collapse of the mortality rate in different countries. We give the values extracted from the collapse of Fig 3B: the increase of the mortality with age (proportional to the uniform fatality ratio $\hat{f}_\alpha$) and the number of infections in each country with respect to the number of infections in Spain $I_\mathcal{C}/I_{\text{Spain}}$ equal to the collapsing constant $\mathcal{D}(\mathcal{C})$. The relative scaling of the mortality above 70 years old is expected to be significantly underestimated. Errors are obtained using the boostrap method at 95% of confidence. The errors of $\mathcal{D}(\mathcal{C})$ are only the statistical errors extracted from the data collapse, they do not include the systematic error associated to the different policies of death counting the different countries which would be much larger, we try to give a better estimate below.
