# Supplementary figures and images for: A scaling approach to estimate the age-dependent COVID-19 infection fatality ratio from incomplete data
Source: PLoS One. 2021 Feb 17;16(2):e0246831. doi: 10.1371/journal.pone.0246831 (PMC7888669; doi:10.1371/journal.pone.0246831)

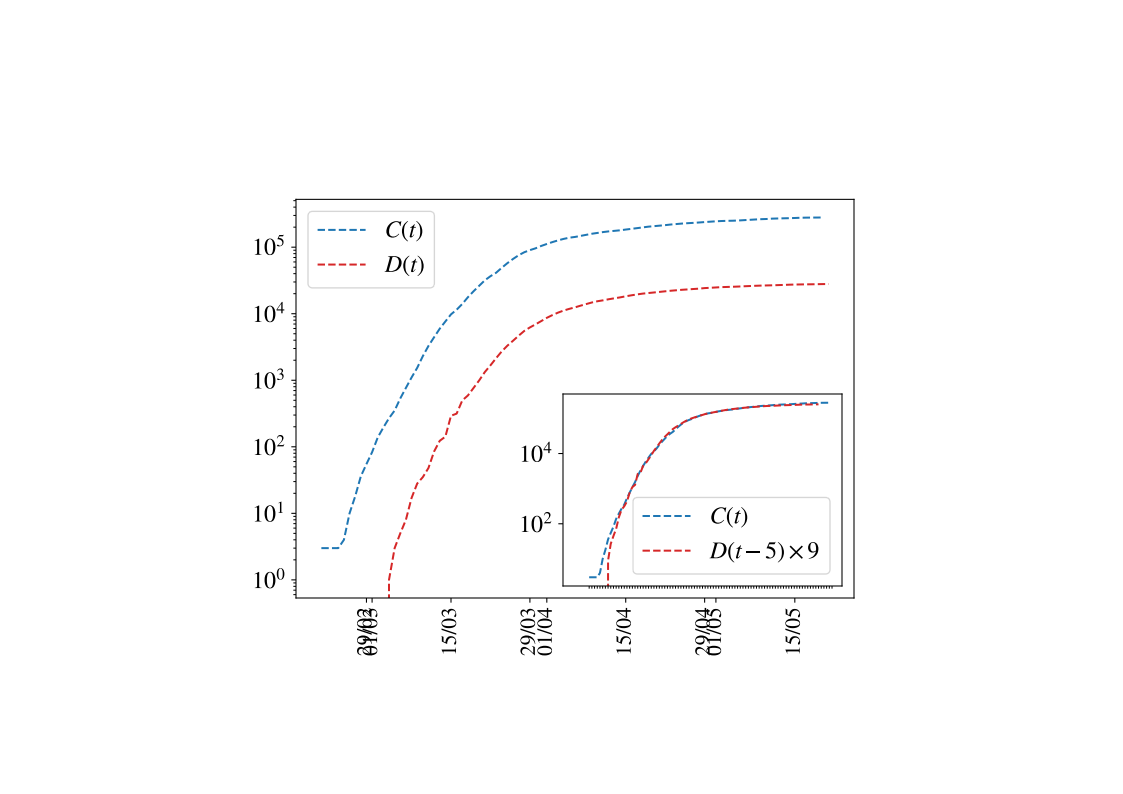

Supplement: S1 Fig — We show the evolution with time of the cumulative total number of official COVID-19 cases and deaths in Spain. In the inset the deaths’ curve is displayed 5 days backwards in time and multiplied by 9, following very precisely the cases’ evolution once it surpassed approximately the 100 cases. Please not that cases are confirmed much later than the infection date and later than the onset of apparition of symptoms. (TIF) [file pone.0246831.s003.tif]

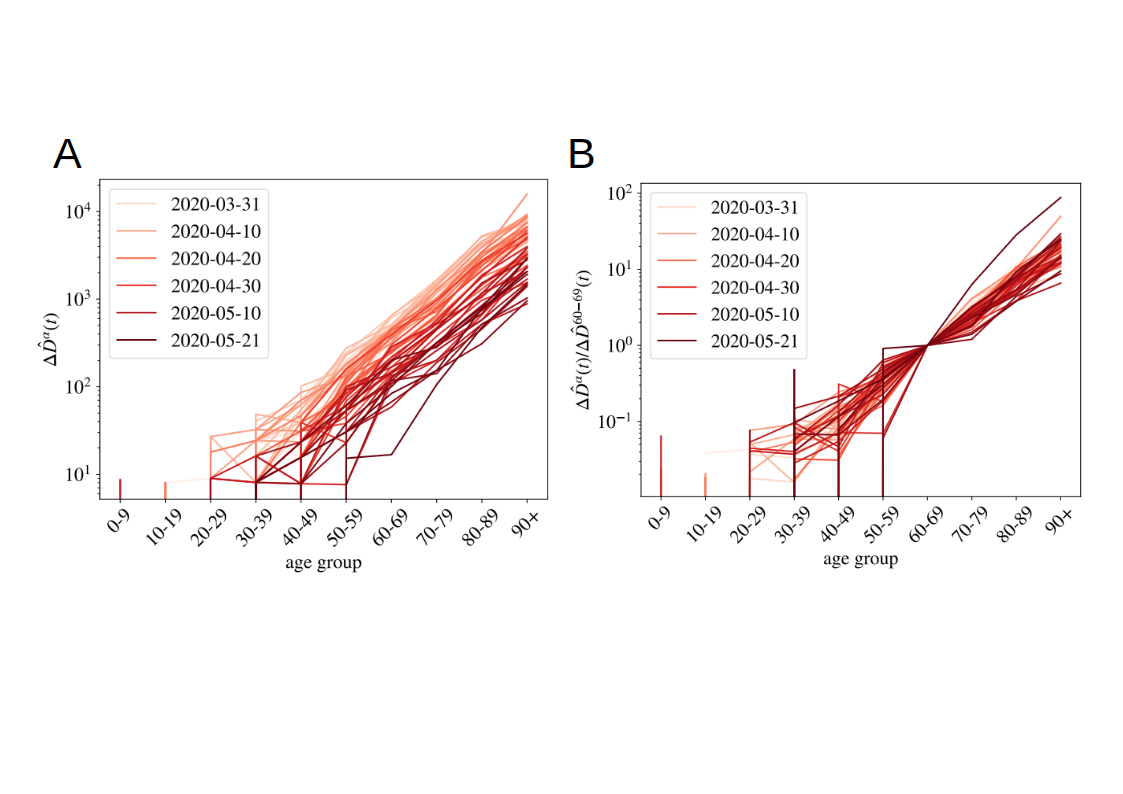

Supplement: S2 Fig — A We show the daily measures of deaths for age group α (normalized by the population density at this group), ΔD^α(t), for different dates. The darker the color, the more recent the measure. In B we show the collapse of the data when we normalize the data with the numbers of group 60-69 years old. Distinct date data collapse worse in a single curve than in the case of the cumulative number of deaths in Fig 1 because being the daily measures smaller, the fluctuations are much larger, yest, we do not observe any systematic change of the attack risk rα with time. (TIF) [file pone.0246831.s004.tif]

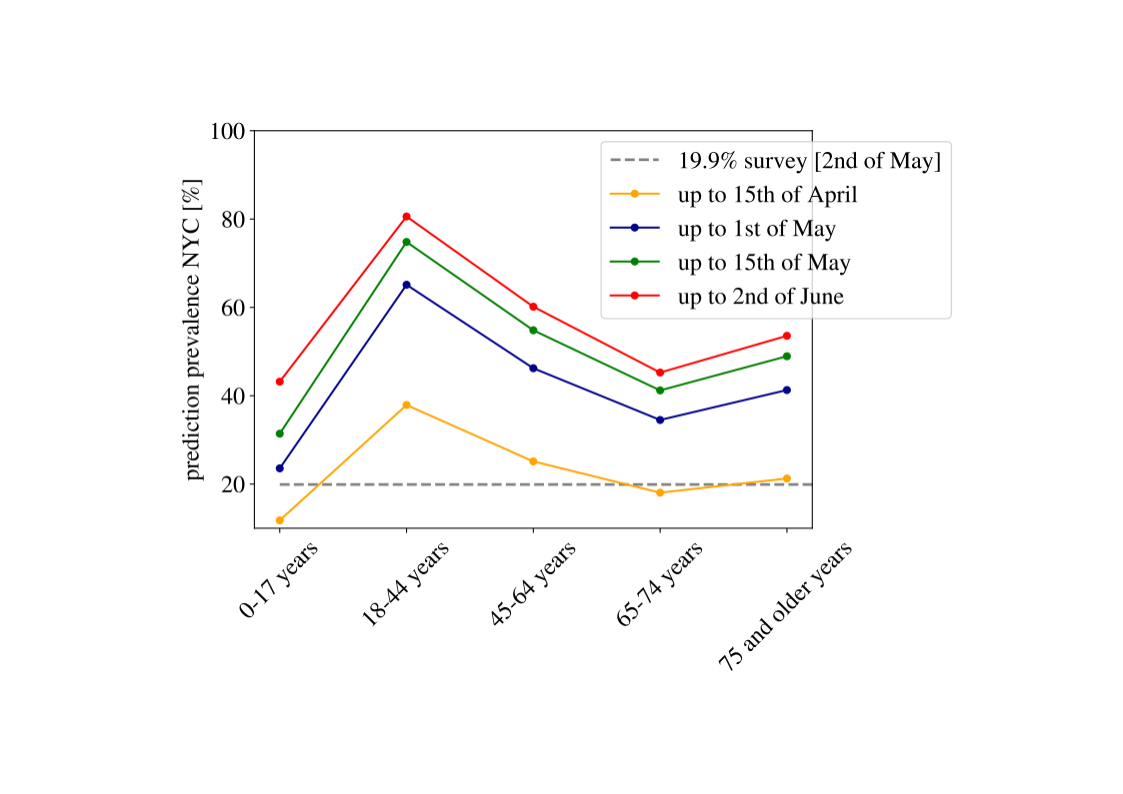

Supplement: S3 Fig — We show our predictions for the sero-prevalence presence in New York City using the death age profile published at different dates and the IFR of Table 1 (without under-counting corrections). Our predictions are significantly higher than the results of the sero-epidemiological survey announced by the New York State Governor the 2nd of May of 2020. (TIF) [file pone.0246831.s005.tif]

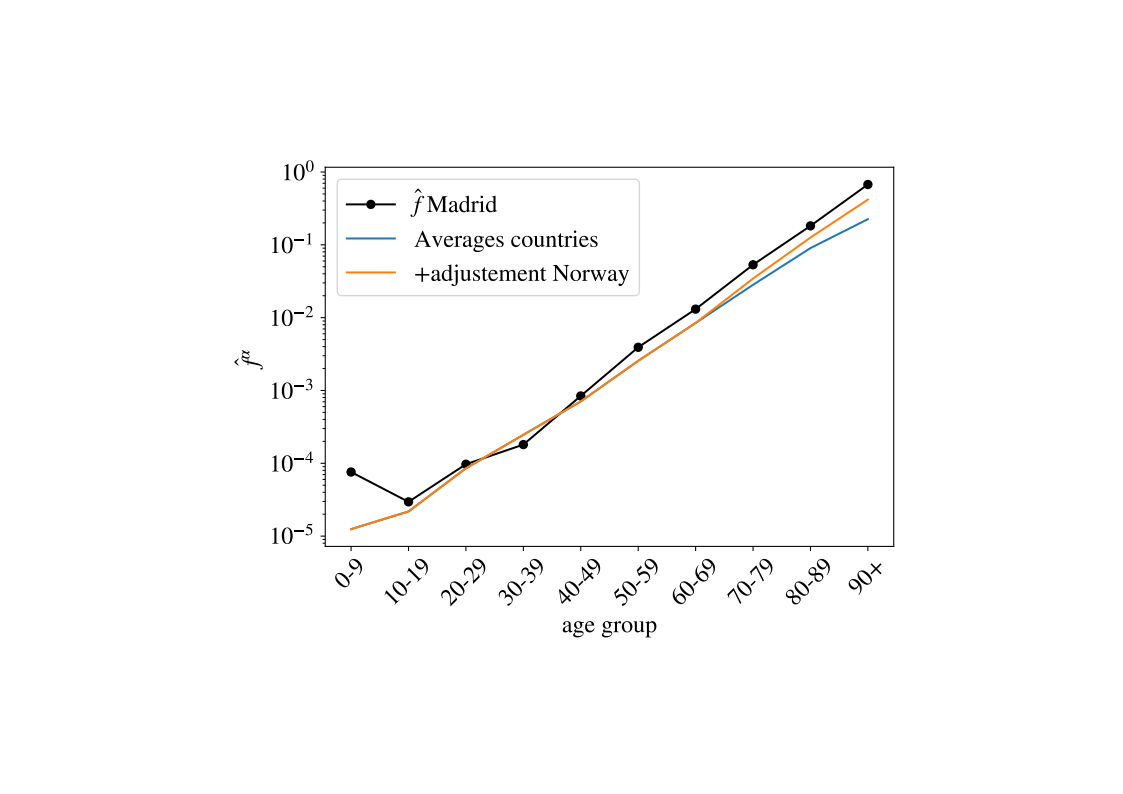

Supplement: S4 Fig — We see that this correction match very well the scaling observed in Madrid’s data. (TIF) [file pone.0246831.s006.tif]

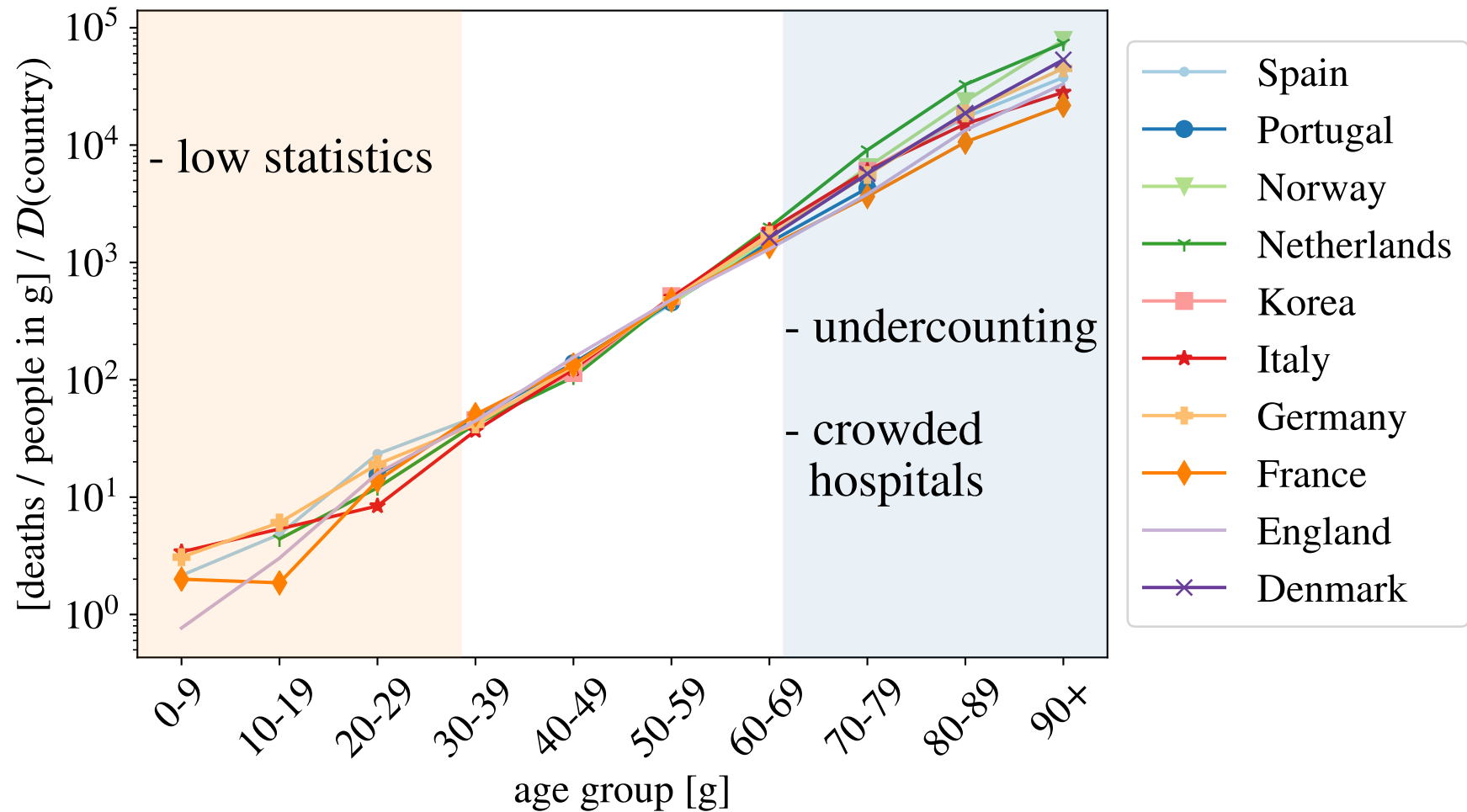

Supplement: S5 Fig — (PDF) [file pone.0246831.s007.pdf]
