# Supplementary material for: A scaling approach to estimate the age-dependent COVID-19 infection fatality ratio from incomplete data
Source: PLoS One. 2021 Feb 17;16(2):e0246831. doi: 10.1371/journal.pone.0246831 (PMC7888669; doi:10.1371/journal.pone.0246831)
Supplement: Attachment — Submitted filename: letter_to_editor_.pdf [file pone.0246831.s009.pdf]

Dear Editor,

After a first round of corrections, both referees agree to the publication of the manuscript "*A scaling approach to estimate the age-dependent COVID-19 infection fatality ratio from incomplete data*" in PLoS ONE in the present format and no additional query was proposed. I take now the chance to thank the referees for their time and useful suggestions.
Finally, Editors suggested to review and include some recent references in the text. I have prepared a new version which includes the reference suggested by the Editor (new ref. 9), and a collection of 9 additional references published on the topic in PLoS ONE (new refs. 7-13, 20, 21 and 41).

I attach the new version of the manuscript that I hope is now found suitable for being published in PloS ONE.

Best regards,
Beatriz Seoane
